# The influence of p02, BE, BB parameters of perinatally obtained cord blood on quantity of obtained cells and focus on the perfect donor criteria

**DOI:** 10.1007/s10561-018-9730-1

**Published:** 2018-10-19

**Authors:** Cecylia Jendyk, Agnieszka Drosdzol-Cop, Agnieszka Nowak-Brzezińska, Marcin Sadłocha, Rafał Stojko, Żaneta Jastrzȩbska-Stojko

**Affiliations:** 1 Primary Maternity Care Department, Faculty of Obstetrics, State Medical College of Higher Education in Opole, Katowicka 68, Opole, Poland; 2 School of Health Sciences in Katowice, Medical University of Silesia, Medyków 12, Katowice, Poland; 3 Institute of Computer Science, Faculty of Computer Science and Material Science, Silesian Uniwersity, Bȩdzińska 39, Sosnowiec, Poland; 4 Department of Anesthesiology and Intensive Care, Medical University of Silesia, Medyków 14, Katowice, Poland

**Keywords:** Stem cell, Umbilical cord blood, Biophysical parameters, Cord blood gas

## Abstract

The aim of the study was to analyze the influence of oxygene partial pressure (*p*02), base excess (*BE*) and buffer base (*BB*) parameters of cord blood obtained perinatally on quantity of obtained cells and focus on the perfect donor criteria. The study included 50 pregnant women aged between 18 and 38 years in which spontaneous labors and elective cesarean sections were performed. Umbilical cord blood was collected immediately after the women were delivered of newborns. The cells were analyzed in the Polish Stem Cells Bank in Warsaw. In the study group of patients different stem cells viability levels did not differ significantly in terms of *pO*2, *BB* and *BE* level, however, there was a trend that the higher the viability the lower *BE* value. The experiment showed also that the cord blood (*CB*) oxygenation scope is vitally important for the *CB* cells viability.

## Introduction

Stem cell (*SC*) has been the object of interest of scientists since the middle of the last century. Its potential and applications are still being discovered and improved. First research works related to stem cells have been carried out on animals and the subject has been most popular at the beginning of the twentyfirst century (Haan and Zant 2002).

In case of SC from cord blood the requirements regarding the histocompatibility of the human leucocyte antigens (HLA) are smaller. Normally, it is required to select 6 antigenes and to match at least 4 of them. At the moment cord blood is used in transplants of unrelated recipients or sibling and in majority of cases it is limited to the haematologic transplantation. The cord blood is a reach source of hematopoietic and progenitor stem cells, which makes it a good alternative source of transplant material in allogeneic transplantations during the treatment of haematological, oncologic and immunological diseases (Pperley et al. 2008; Wożniak 2003; Akalin et al. 2001; Turner et al. 2013).

Almost unlimited potential of stem cell application in transplantology has been noticed also in Poland. In 2010 a team of experts from the Polish Gynaecological Society issued a favourable opinion on “the collection and storage of the umbilical cord blood stem cells” that supports the idea of collection and storage of umbilical cord blood in specialised banks for further medical use (Porȩba et al. 2010).

Cord blood from each perinatal collection intended for storing in blood bank is tested for the count and viability of nucleated and $CD 34+$ cells. Data from this analysis determine the properties of each blood sample and its utility for transplantation. In public cord blood banks each sample is also analysed for human leukocyte antigen (HLA) system. It is done to better match a donor and a recipient and allows for using the sample in case of transplant between unrelated patients (Ołdak et al. 2000).

The blood sample preparation includes a number of processes. Its aim is to reduce samples volume by spinning in a concentration gradient or by sedimentation method with application of HES (hydroxyethyl starch). Before freezing a suspension of cells is mixed with cryoprotector and cooled to $-\,80^{\circ }$ in a device for controlled freezing, before placing it in cryostats in temperature from $-\,125^{\circ }$ to $-\,196^{\circ }$. Data regarding samples used for transplantation confirm their utility even after 15–24 years from collection (Machaj et al. 2001; Burgio et al. 2003; Broxmeyer 2010).

Umbilical cord blood gas testing results are vital for the evaluation of new-borns well-being after birth. Many authors agree that the threshold value for foetal anoxia is $pH \le 7.1$. Except for *pH* and *pCO*2 parameters the *SC* properties are determined also by oxygen partial pressure (*pO*2) and bases shortage (*BE*, *BB*) (Pomorski et al. 2010; Mancinelli et al. 2006). Scientific reports regarding the cord blood gas testing values in reference to the mode of delivery are inconclusive. Many scientific papers indicate significant differences in carbon dioxide concentration values in umbilical cord blood (Richardson et al. 2005; Redzko et al. 2005).

## Aim of the paper

Many factors, which could influence the quality of cell material are taken into account at the moment. Some of the factors taken into consideration are: storage time, preparation times, anticoagulant used or age of the patients taking part in the blood storage in core blood banks (Gessler and Dahinde 2003).

The aim of this paper was to show the relationship between bio-physical parameters (*pO*2, *BE* and *BB*) of umbilical cord blood and the quality of the collected material, based on the material from donors accepted for the study group.

The aim of this paper was also to determine the impact of *pO*2, *BB* and *BE* level in the umbilical cord blood on the $CD 34+$ cells and white blood cells (*WBC*) count, as well as to determine the vitality of cord blood SC and to show the relationship between the mode of delivery and the quality of the collected material.

## Material and methods

The research covered 50 pregnant women hospitalised in the obstetric- gynaecological ward with the oncological gynaecology department of the *Boni Fraters Hospital* in *Katowice* from 8th February to 30th September 2012, whose pregnancy was terminated with vaginal or caesarean birth. The research included healthy patients aged 18 to 38 without any deviations from physiological condition during the pregnancy. Inclusion criterion was the informed consent of the patient for the participation in research project, consistent with the protocol accepted by *the Bioethical Committee of the Medical University of Silesia* issued on 7th February 2012.

34 out of 50 patients terminated the pregnancy with vaginal birth without complications and in case of 16 patients the pregnancy was terminated with caesarean birth before dilation contractions appeared. Umbilical cord blood was collected right after birth, and after closing and severance of the cord, both in case of vaginal and caesarean birth. The material was collected by puncturing the cords veins and gathering material to a container with CPD (Citrate Phosphate Dextrose Solution) anticoagulant in the quantity of approx. 1–2 ml. Test tubes were placed in special stabilising gel and transported to the lab of the *Polish Bank of Stem Cells* in *Warsaw*. Preparation of the umbilical cord blood consists of reducing the volume of cord blood by eliminating red blood cells and part of autologous plasma. Red blood cells were eliminated by sedimentation in hydroxyethyl starch environment (HES 6% solution, Grifols), and plasma was eliminated by supernatant spinning (625×*g*, 15 min, 20°).

The number of nucleated cells was evaluated based on the test results conducted in haematology analyser MICROS 60 (Horiba ABX). The evaluation was done twice: before and after sample preparation. The percentage of cells recovery was estimated based on both results.

Viability of cells in the umbilical cord blood sample was established based on the fluorescence level of the 7-AAD labelled cells (Via Probe, BD) in accordance with the producer’s recommendations. After labelling, cells were analysed with the use of flow cytometry equipment (FACSCalibur, program CellQUEST Pro, BD). The amount of $CD 34+$ and $CD 45+$ phenotype cells was calculated after labelling the suspension of cells with monoclonal antibodies conjugated with phycoreythrin directed against HPCA-2 antigens and monoclonal antibodies conjugated with fluorescein (FITC) directed against CD45 antigen. Isotopic control was provided by cells labelled with appropriate polyclonal antibodies. The percentage of haematopoietic progenitor cells was established after the flow cytometric analysis, and their total count on the basis of their percentage and the number of nucleated cells established with haematology analyser.

Second fraction of umbilical cord blood was collected to special heparinized syringe in the quantity of approx. 0.5 ml and its *pO*2, *BE* and *BB* acid–base balance was determined within 5 min with pH-meter Cobas 121. The cord blood collection took place before the third stage of labour finished.

The results were analysed with *MS Office Excel spread-sheet* and *Statistica PL software*. Statistical analysis of the collected data was done with the application of descriptive, graphic (graphs) and statistical analysis methods. Average value is the *arithmetical mean* and the statistical dispersion is the *standard deviation (SD)*. From the available statistical analysis methods the analysis of variance (*ANOVA*) was selected. Moreover, data mining was carried out with the use of decision trees and cluster analysis with free R package. Level of $p<0.05$ was adopted as statistically significant (Stanisz 2006, http://cran.r-project.org/, http://rattle.togaware.com/).

## Results

In the analysed group of 50 patients 32% of births were caesarean births (16 patients) and 68% were vaginal births (34 patients).

The abbreviation *Ns* in all tables in a cell with the value *p* means no statistical significance for the parameter being tested.

Due to the fact that the basis for analysis were *pO*2, *BE* and *BB* concentration values, Table 1 shows the basic characteristics of these features. Among 50 patients 90% had acidosis and only 10% alkalosis (60% of patients had *BB* concentration below the norm and 40% within the norm). Average *BB* concentration was $44.52\pm 2.52$, while average *BE* concentration was $-\,3.85\pm 2.33$ and *pO*2 average concentration was $25.49\pm 7.12$.

**Table 1:** The basic characteristics of pO2, BE, BE

	Mean ± SD	Min–max
Age	$28.86\pm 4.54$	20.0–38.0
Number of pregnancy	$1.56\pm 0.81$	1.0–4.0
Number of birth	$1.48\pm 0.79$	1.0–4.0
*po*2	$25.49\pm 7.12$	13.3–45.6
*BE*	$-\,3.85\pm 2.33$	$-\,9.0{-}0.4$
*BB*	$44.52\pm 2.52$	39.5–48.4
BB	Acidosis, n = 45	90%
	Alkalosis, n = 5	10%
BB	Below norm, n = 30	60%
	Within the norm, n = 20	40%

One of the main aims of this paper was to examine the impact of *pO*2, *BB* and *BE* of the umbilical cord blood collected during delivery on the quality of the material. Therefore, correlation analysis (chi square test) was performed with the use of suitable statistical tools (Table 2).

**Table 2:** The correlation analysis between the po2, BE, BB and CD34+ and cells viability

	CD34+	Cells viability
*po*2 (mmHg)	$r=-\,0.0959, p=0.526$	$r=-\,0.1065, p=0.481$
*BE* (mmol/l)	$r=0.0727, p=0.631$	$r=-\,0.1169, p=0.439$
*BB* (mmol/l)	$r=0.0227, p=0.881$	$r=-\,0.0869, p=0.566$

No statistically significant correlation was established. At the same time it is visible that the correlation of *pO*2 and the percentage of stem cells or their viability is negative, which means that as *pO*2 condensation increases, the percentage or viability of stem cells decreases. In case of *BE* and *BB* there is a positive correlation with the percentage of $CD34+$ cells and negative correlation with cells viability.

In the analysis of the collected umbilical cord blood the average viability of *SC* was on the level of 97.15. the lowest value was recorded in case of vaginal births and equalled 86.71. the highest value of 99.88 was recorded in a blood sample collected during caesarean birth, with *pH* on the level of 7.25. In case of 48% of patients the viability of cells was the highest ($>98$) and for 20% in each group cells viability was 95–97 and 97–98. Only 10% had cells viability in the range of 90–95 and only 2% had cells viability $<90$.

Taking into account the $CD34+$ cells count it is visible that 8% of patients showed CD34+ count below 0.1. Majority of patients (82%) had CD34+ count in the scope of 0.1–0.5. The $CD34+$ count above 0.5 was recorded only with 10% of patients. We should also note that for majority of patients in both groups the viability of cells was above 95. From the tested samples only in case of 20% of patients WBC were within the standard scope ($4{-}10\,\hbox {k}/\upmu \hbox {l}$), and 80% were above $10\,\hbox {k}/\upmu \hbox {l}$. The average WBC count in the material was $13.84\,\hbox {k}/\upmu \hbox {l}$, minimum $7.8\,\hbox {k}/\upmu \hbox {l}$ and maximum $27.20\,\hbox {k}/\upmu \hbox {l}$. For majority of patients (78%) the WBC count did not exceed $16.20\,\hbox {k}/\upmu \hbox {l}$.

Further, the percentage of vaginal and caesarean births for each group was analysed. For 89% of patients with WBC count $>15\,\hbox {k}/\upmu \hbox {l}$ had vaginal birth, and only 11% had caesarean birth. Majority of patients (63%) with WBC count $10{-}15\,\hbox {k}/\upmu \hbox {l}$ had vaginal birth, and in case of standard WBC count ($4{-}10\,\hbox {k}/\upmu \hbox {l}$) majority of patients (60%) had caesarean birth.

The paper assumed that *pO*2, *BE* and *BB* might influence the stem cells viability and the $CD34+$ cells count. In the study group of patients it was showed that different stem cells viability levels did not differ in a statistically significant way in terms of *pO*2, *BE* and *BB* levels. Although there was a trend that higher cells viability can be related to lower *BE* value, but the difference was not statistically significant and the test would have to be repeated on bigger study group (Table 3).

**Table 3:** The influence of po2, BE , BB on the stem cells viability and the $CD34+$ cells count

Cells viability	pO2	BE	BB
	$\hbox {Mean}\pm \hbox {SD}$ (mmHg)	$\hbox {Mean}\pm \hbox {SD}$ (mmol/l)	$\hbox {Mean}\pm \hbox {SD}$ (mmol/l)
90–95	$21.88\pm 7.11$	$-\,3.03\pm 1.73$	$45.08\pm 1.82$
95–97	$32.61\pm 25.87$	$-\,3.12\pm 1.62$	$45.00\pm 2.07$
97–98	$25.87\pm 5.64$	$-\,3.81\pm 2.23$	$44.91\pm 2.50$
$>98$	$26.09\pm 8.50$	$-\,4.28\pm 2.67$	$44.11\pm 2.81$
Total	$26.93\pm 13.08$	$-\,3.85\pm 2.33$	$44.52\pm 2.52$
*p*	Ns	Ns	Ns

While analysing the above table we may notice, that patients with cells viability above 98% have the highest average shortage of *BE* and *BB* bases. It was also visible that despite highest average *pO*2 (32.61 mmHg) the stem cells viability was on the level of 95–97%. This data suggest that the umbilical cord blood oxygenation level is crucial for stem cells viability.

During the next stage of research the $CD34+$ cells count was estimated for different acid–base balance parameters (Table 4). The level of *pO*2, *BE* and *BB* for patients with different $CD34+$ cells percentage was not statistically significant. Although, there was a trend that the higher *pO*2 the higher percentage level of $CD34+$ cells, but the differences were not statistically significant. There was no such tendency for *BE* and *BB*.

**Table 4:** The level of *pO*2, *BE* and *BB* for patients with different $CD34+$ cells percentage

CD34+	pO2	BE	BB
	$\hbox {Mean}\pm \hbox {SD}$ (mmHg)	$\hbox {Mean}\pm \hbox {SD}$ (mmol/l)	$\hbox {Mean}\pm \hbox {SD}$ (mmol/l)
0.01–0.09	$45.30\pm 37.14$	$-\,4.10\pm 0.92$	$45.15\pm 1.06$
0.1–0.2	$24.18\pm 7.26$	$-\,4.05\pm 2.53$	$44.33\pm 2.70$
0.2–0.3	$25.26\pm 5.57$	$-\,4.18\pm 2.38$	$43.93\pm 2.54$
0.3–0.4	$25.70\pm 14.44$	$-\,1.33\pm 1.27$	$46.90\pm 1.24$
0.4–0.5	$28.00\pm 3.80$	$-\,3.17\pm 2.20$	$45.40\pm 2.88$
$>0.5$	$27.80\pm 8.04$	$-\,4.97\pm 2.37$	$43.33\pm 2.73$
Total	$26.93\pm 13.08$	$-\,3.85\pm 2.33$	$44.52\pm 2.52$
	Ns	Ns	Ns

It was decided to estimate if there were significant differences of the acid–base balance of the umbilical cord blood in case of vaginal and caesarean birth. Taking into account *pO*2, *BE* and *BB* levels in case of patients after vaginal and caesarean birth, it was visible that the patients after vaginal birth had higher *BE* deficit value in statistically significant way than patients after caesarean birth. The result was similar in case of *BB*. Patients after vaginal birth had lower *BB* level in statistically significant way than patients after caesarean birth (Table 5).

**Table 5:** *pO*2, *BE* and *BB* levels in case of patients after vaginal and caesarean birth

Birth type	pO2	BE	BB
	$\hbox {Mean}\pm \hbox {SD}$ (mmHg)	$\hbox {Mean}\pm \hbox {SD}$ (mmol/l)	$\hbox {Mean}\pm \hbox {SD}$ (mmol/l)
Vaginal birth	$28.72\pm 15.46$	$-4.66\pm 2.22$	$43.80\pm 2.35$
Caesarean birth	$23.48\pm 5.28$	$-2.34\pm 1.76$	$45.89\pm 2.29$
Total	$26.93\pm 13.08$	$-\,3.85\pm 2.33$	$44.52\pm 2.52$
*p*	Ns	*p* = 0.000803	*p* = 0.005852

While monitoring patients for *BE* levels it can be noticed that patients with *BE* below norm ($\pm \,2.3$) had lower $CD34+$ cells concentration percentage and lower cell viability than patients with *BE* within the norm (Table 6). However, this differences were not statistically significant and it would be necessary to make trials on a larger group.

**Table 6:** The $CD34+$ cells concentration percentage and cell viability for patients with different *BE* levels

BE	CD34+	$CD34+\%$	Cells viability
	$\hbox {Mean}\pm \hbox {SD}$	$\hbox {Mean}\pm \hbox {SD}$	$\hbox {Mean}\pm \hbox {SD}$
	Min–max	Min–max	Min–max
Below norm	$110.40\pm 73.77$	$0.23\pm 0.14$	$97.18\pm 2.56$
	10.00–316.00	0.03–0.63	86.71–99.65
Within the norm	$123.69\pm 58.73$	$0.25\pm 0.12$	$97.32\pm 1.95$
	49.00–252.00	0.10–0.50	92.88–99.88
Total	$115.02\pm 68.54$	$0.23\pm 0.13$	$97.22\pm 2.35$
	10.00–316.00	0.03–0.63	86.71–99.88
*p*	Ns	Ns	Ns

Determining *BE* parameters for acidosis or alkalosis of the cord blood sample, the patients with acidosis showed lower *CD*34 concentration, $CD34+$ percentage and cells viability than patients with alkalosis. However, the differences were not statistically significant and should be repeated on bigger study group (Table 7).

**Table 7:** The $CD34+$ cells concentration percentage and cell viability for patients with acidosis or alkalosis

BE	CD34+	$CD34+$%	Cells viability
	$\hbox {Mean}\pm \hbox {SD}$	$\hbox {Mean}\pm \hbox {SD}$	$\hbox {Mean}\pm \hbox {SD}$
	Min–max	Min–max	Min–max
	(Median)	(Median)	(Median)
	Q1–Q3	Q1–Q3	Q1–Q3
Acidosis	$113.4\pm 68.42$	$0.23\pm 0.13$	$97.18\pm 2.35$
	10.0–316.0	0.03–0.63	86.71–99.88
	(97.0)	(0.19)	(97.86)
	70.0–121.0	0.16–0.24	96.28–98.35
Alkalosis	$177.0\pm 104.81$	$0.35\pm 0.21$	$96.87\pm 1.95$
	91.0–352.0	0.18–0.70	94.21–99.23
	(147.0)	(0.29)	(97.11)
	107.0–188.0	0.21–0.38	95.76–98.02
*p*	Ns (0.0676)	Ns (0.0777)	Ns (0.7758)

## Discussion

There are high hopes for stem cells. The Nobel Prize for 2012 was awarded for research on the possibility of differentiation of stem cells in any pre-programmed way (Gurdon 1962). Scientists managed to trigger involution of mature cells by inverting their development and it has also been noted that these cells start to divide and can give the beginning to any cell of the body (Takahashi and Yamanaka 2006). Mature cell can be reverted to stem cell and next turned into any body cell. Development of genetics, biology and regenerative medicine creates possibilities for better diagnosis and for the use of new, more efficient treatment methods. Scientists still work on stem cells, trying to prompt them to differentiate to specific cell types, without turning into cancer cells. Mayanai and his team demonstrated that umbilical cord blood stem cells usually do not contain cancer cells and have fewer acquired genetic defects (Mayani and Velez-Ruelas 2003). Works on stem cells multiplication methods, improving the conditions and medium for growth and proliferation stimulation factors are still being carried out. Ex vivo growth allows for multiplication of haematopoietic progenitor stem cells for treatment and experiments.

The research compering different modes of delivery aimed to evaluate differences in umbilical cord blood arterial blood gas, the reaction of white blood system and endocrine system, as the indicator for foetus wellbeing. M. Pomorski and his team in their research did not find any significant pH difference during delivery and during caesarean birth. Earlier research of Mancinelli and his team showed significantly higher *WBC* count in the umbilical cord blood after vaginal birth (Pence et al. 2002; Irested et al. 1982). The priority in stem cells collection is to obtain the best transplantation material. Many factors that could impact the quality of the potential biological material are taken into consideration. These are patients age, storage, transport and preparation temperature, anticoagulant type, course of the birth and the mode of delivery.

This research aimed at determining the impact of umbilical cord blood *pO*2, *BB* and *BE* value on the quality of transplantation material. The paper assumed that the above factors may have influence on the viability of stem cells and on the percentage of $CD34+$ cells. In the study group of patients different stem cells viability levels did not differ significantly in terms of *pO*2, *BB* and *BE* level, however, there was a trend that the higher the viability the lower *BE* value.

The experiment showed also that despite of the highest average *pO*2 level (32.61 mmHg) viability of stem cells was on the level of 95–97%. This suggests that the CB oxygenation scope is vitally important for the cord blood cells viability.

The next stage of research determined the $CD34+$ cells count in the samples of different acid–base balance parameters. It was visible that the higher *pO*2 concentration the higher is the $CD34+$ cells percentage, however, the differences are not statistically significant. This tendency was not visible in correlation with *BB* or *BE*. It has been decided to determine if the changes of acid–base parameters of CB for women after vaginal and caesarean births are significant. While analysing the *pO*2, *BB* and *BE* level for patients after vaginal and caesarean birth it was established that patients after vaginal birth had higher *BE* deficit value in statistically significant way than patients after caesarean birth. In case of *BB* the value was similar while compering cord blood parameters for *BE* it was noticed that patients with *BE* below the norm ($\pm \,2.3$) had lower $CD34+$ cells concentration percentage and lower SC viability than patients with BE within the norm. Further the *BE* parameters were determined for acidosis and alkalosis of the CB samples and the results for patients with acidosis showed lower $CD34+$ concentration, $CD34+$ percentage, and cells viability than for patients with alkalosis. This differences however were not statistically significant and the research should be repeated on bigger study group.

## Conclusions

1. No significant correlation between the mode of delivery and cord blood *SC* viability was observed.
2. Low *BE* values of umbilical cord blood are associated with high *SC* viability.
3. High *pO*2 values of umbilical cord blood are not associated with the highest *SC* viability, no significant correlation of *pO*2 with *SC* viability was confirmed.
4. *BE* and *BB* values of the umbilical cord blood are statistically lower after vaginal births than after caesarean births.
